# Mycoheterotrophy and plastid genome evolution in the early-diverging epidendroid orchid tribe Nervilieae: independent transitions in *Epipogium* and *Stereosandra*

**DOI:** 10.1093/aobpla/plag002

**Published:** 2026-01-16

**Authors:** Craig F Barrett, Cameron W Corbett, Samuel V Skibicki, Vincent S F T Merckx, Matthew C Pace, Paul M Peterson

**Affiliations:** Department of Biology, West Virginia University, 5218 Life Sciences Building, 53 Campus Drive, Morgantown, WV 26506, United States; Department of Biology, West Virginia University, 5218 Life Sciences Building, 53 Campus Drive, Morgantown, WV 26506, United States; Department of Biology, West Virginia University, 5218 Life Sciences Building, 53 Campus Drive, Morgantown, WV 26506, United States; Naturalis Biodiversity Center, Darwinweg 2, Leiden 2333 CR, The Netherlands; Institute of Biology Leiden, Leiden University, Sylviusweg 72, Leiden 2333 BE, The Netherlands; New York Botanical Garden, 2900 Southern Boulevard, Bronx, New York, NY 10458, United States; Department of Botany, National Museum of Natural History, Smithsonian Institution, 1000 Madison Drive Northwest, Washington, DC 20560-0166, United States; Evolution & Diversity

**Keywords:** Orchidaceae, plastome, selection, pseudogene, coalescent, phylogeny, phylogenomics, ancestral state reconstruction

## Abstract

Parasitic organisms are of interest in evolutionary biology, often displaying drastic modifications in morphology, physiology, genomes, and ecology. These properties, however, make them challenging from a systematics perspective. Mycoheterotrophy, in which plants become non-photosynthetic parasites on fungi, is an excellent example, and this unique life history has evolved numerous times in the orchid family. Here, we focused on *Stereosandra*, a genus of mycoheterotrophic orchid comprising a single species, *S. javanica*, about which little is known. *Stereosandra* has been placed in the orchid tribe Nervilieae, along with the leafy, autotrophic *Nervilia*, and the leafless, mycoheterotrophic *Epipogium*. We characterized the first complete plastid genome for *Stereosandra* and used nuclear sequence capture to determine its relationships within Nervilieae. This study presents the first genetic data ever produced for *Stereosandra*. The plastid genome exhibits rampant gene losses, pseudogenes, and reduced size relative to *Nervilia* but not to the extent seen in *Epipogium*. There is evidence of relaxed negative selection in six genes in *Stereosandra*, including *matK*, which functions in Group IIA intron removal of seven plastid genes, four of which have been lost or pseudogenized in this species. Applying mixture models, plastid genomes provided weak support for a sister position of *Stereosandra* to a clade of *Epipogium* + *Nervilia*. Nuclear phylogenomic analyses provided strong support for the same relationships. Ancestral state reconstruction revealed clear evidence that mycoheterotrophy evolved multiple times in the tribe from leafy ancestors. This study provides a previously unidentified, convergent instance of the evolution of full mycoheterotrophy in plants. We discuss the results in the context of proposed models of reductive plastid genome evolution and the genomic and evolutionary consequences of radical life history shifts in heterotrophic plants.

## Teaser

Leafless, parasitic plants are among the most fascinating species, and about half of these occur within the orchid family. Yet, they are among the most difficult to place from a taxonomic perspective. Here we focused on Stereosandra javanica, a poorly collected and little-understood orchid native to Asia, using material sampled from herbaria and high-throughput plastid and nuclear sequencing. We found strong evidence that that Stereosandra represents an independent transition to non-photosynthetic parasitism in the orchids and conclude that the number of independent losses of photosynthesis may be underestimated. We discuss our findings considering recent models that characterize the transition from photosynthesis to photosynthetic loss from a genomic perspective in leafless plants.

## Introduction

Parasites have long been important subjects in evolutionary biology (Price 1980, Poulin 2007). They often display drastic modifications in morphology, genomes, physiology, behaviour, and ecological interactions, driven by evolutionary pressures to exploit their hosts (Nickrent and Musselman 2004, Poulin 2007). The properties that make them fascinating from ecological and evolutionary perspectives also pose significant challenges to their phylogenetic placement and taxonomic status (Nickrent et al. 1998, Keeling and Fast 2002, Corradi and Slamovits 2011). These include reduction in morphological, genomic, and reproductive features, which translate to a general lack of character information available for their placement, and elevated genomic substitution rates associated with modified selective pressures, leading to issues such as long branch attraction and genomic deletions. Mycoheterotrophy is a prime example, in which plants parasitize fungi and is estimated to have evolved over forty times independently across plants (Leake 1994, Bidartondo 2005, Merckx and Freudenstein 2010, Merckx 2013, Waterman et al. 2013, Merckx et al. 2013a, 2013b).

Over thirty independent transitions to heterotrophy are hypothesized to have occurred within a single, megadiverse plant family, Orchidaceae, providing a rich source of independent case studies, and making orchids a powerful group to test hypotheses on the consequences of such evolutionary shifts (Freudenstein and Barrett 2010, Merckx et al. 2013a, 2013b, Barrett et al. 2019, Ogura-Tsujita et al. 2021). The unique life history in orchids, termed initial mycoheterotrophy, involves seeds that contain no endosperm and require fungi for germination, followed by an obligately parasitic protocorm stage in which the seedling is completely dependent on fungal nutrition (Leake 1994, Rasmussen 1995, Bidartondo 2005, Taylor et al. 2013, Rasmussen et al. 2015). Initial mycoheterotrophy may pre-adapt orchids for shifts to heterotrophy at maturity; partial mycoheterotrophs retain some level of photosynthesis but depend on varying amounts of fungal-derived nutrition, whereas in full mycoheterotrophy photosynthesis is lost, and the relationship is completely parasitic. Leaf laminae (and sometimes roots) are typically lost or reduced, representing apparent cases of convergent evolution in growth form. Full mycoheterotrophy and the associated loss of photosynthesis represent non-reversible evolutionary transitions, and as a result mycoheterotrophic plants have been the subjects of continued ecological, genomic, and physiological study (Gebauer and Meyer 2003, Julou et al. 2005, Hynson et al. 2013, Merckx 2013, Merckx et al. 2013b).

Plastid genomes (plastomes) have been a major focus of phylogenetic and evolutionary studies in parasitic plants, including mycoheterotrophic orchids, and have been particularly informative on the transition to holoparasitism and full mycoheterotrophy (Barrett et al. 2014, Wicke et al. 2016, Graham et al. 2017). Various conceptual models have been proposed to describe the patterns of gene loss and pseudogenization in heterotrophic plants, ranging from autotrophy to full mycoheterotrophy or holoparasitism, with the latter two conditions nearly always displaying functional gene loss in photosynthesis-related gene classes (Wicke et al. 2011, 2013, 2016, Barrett and Davis 2012, Barrett et al. 2014, 2019, Graham et al. 2017). In more ‘advanced’ parasites the plastome is reduced to a fraction of that in autotrophs, with losses of so-called ‘housekeeping’ genes (Molina et al. 2014, Schelkunov et al. 2015, Bellot and Renner 2016, Wicke et al. 2016, Klimpert et al. 2022).

Barrett and Davis (2012), Barrett et al. (2014), and Wicke et al. (2016) proposed ‘staged’ models in which gene functional classes are lost roughly in the following sequence: (i) *ndh genes,* encoding the NAD(P)H Dehydrogenase complex, which functions in electron cycling under variable light conditions; (ii) genes directly involved in the photosynthetic machinery (*psa, psb, pet*), encoding Photosystems I, II, and Cytochrome b_6_/f complexes, respectively; (iii) photosynthesis-associated genes with other putative functions (*atp*, *rbcL*), encoding the ATP Synthase complex and RuBisCO Large Subunit, respectively; (iv) ‘housekeeping’ genes involved in basic organellar functions such as intron removal, fatty acid biosynthesis, protease activity, and translation (e.g. *accD*, *clpP, matK*, *rpl*, *rps*); and (v) complete loss of the plastome. Graham et al. (2017) subsequently presented a model with more relaxed stage boundaries, favouring a more continuous or idiosyncratic trajectory of losses among lineages, but with the added observation that five ‘core non-bioenergetic’ genes tend to be among the last to be lost (*accD*, *clpP*, *trnE^UUC^*, *ycf1*, and *ycf2*).

While fascinating from an evolutionary perspective, plastid genomes in parasites may be prone to long branch attraction in phylogenetic analyses due to gene losses and highly accelerated substitution rates (Lemaire et al. 2011, Bromham et al. 2013, Lam et al. 2018, Garrett et al. 2023, Barrett et al. 2024). The heavy reliance on plastid DNA in orchid systematic studies thus may not be informative for the placement of the many mycoheterotrophic orchid lineages (e.g. Chase et al. 1993, 2015, Cameron et al. 1999, Freudenstein and Chase 2015, Barrett et al. 2024, Freudenstein 2024).

The total number of times full mycoheterotrophy has evolved in (extant) orchids is unknown, but the current hypothesis of 30 such transitions may be an underestimate (Freudenstein and Barrett 2010, Merckx and Freudenstein 2010, Merckx et al. 2013a, Barrett et al. 2014, Chase et al. 2015, Barrett et al. 2019, Gomes et al. 2019, Ogura-Tsujita et al. 2021, Freudenstein 2024). The species-rich orchid subfamily Epidendroideae Lindl. ex Endl., with 16 tribes, over 600 genera, and an estimated 21 800 species, contains the majority of fully mycoheterotrophic taxa (Pridgeon et al. 2009, Chase et al. 2015). This is especially the case among several of the tribes comprising the epidendroid ‘base’, or Early Diverging Epidendroideae (EDE), a non-monophyletic assemblage of eight tribes that are predominantly terrestrial in habit. The other eight tribes (here referred to as the Core Epidendroideae, or CE), by contrast, are largely epiphytic, and together are supported as monophyletic, representing the bulk of orchid species diversity (Dressler 1993, Pridgeon et al. 2009, Collobert et al. 2023, Freudenstein 2024). Among the EDE tribes, Gastrodieae Lindl. (*Auxopus* Schltr.*, Didymoplexiella* Garay*, Didymoplexis* Griff*., Gastrodia* R. Br., and *Uleiorchis* (POWO 2025) are exclusively composed of fully mycoheterotrophic species and represent approximately 25% of all mycoheterotrophic plant diversity. Fully mycoheterotrophic species are also found in tribes Wullschlaegelieae Dressler (*Wullschlaegelia* Rchb. f.), Neottieae Lindl. (*Apyllorchis* Blume, *Cephalanthera* Rich., and *Neottia* Guett.), Triphoreae Dressler (*Pogoniopsis* Rchb. f.), Nervilieae Dressler (*Epipogium* J.F. Gmel. ex Borkh. and *Stereosandra* Blume), and Tropidieae Dressler (some species of *Tropidia* Lindl.) (Merckx et al. 2013a, Freudenstein 2024).

**Figure 1:**
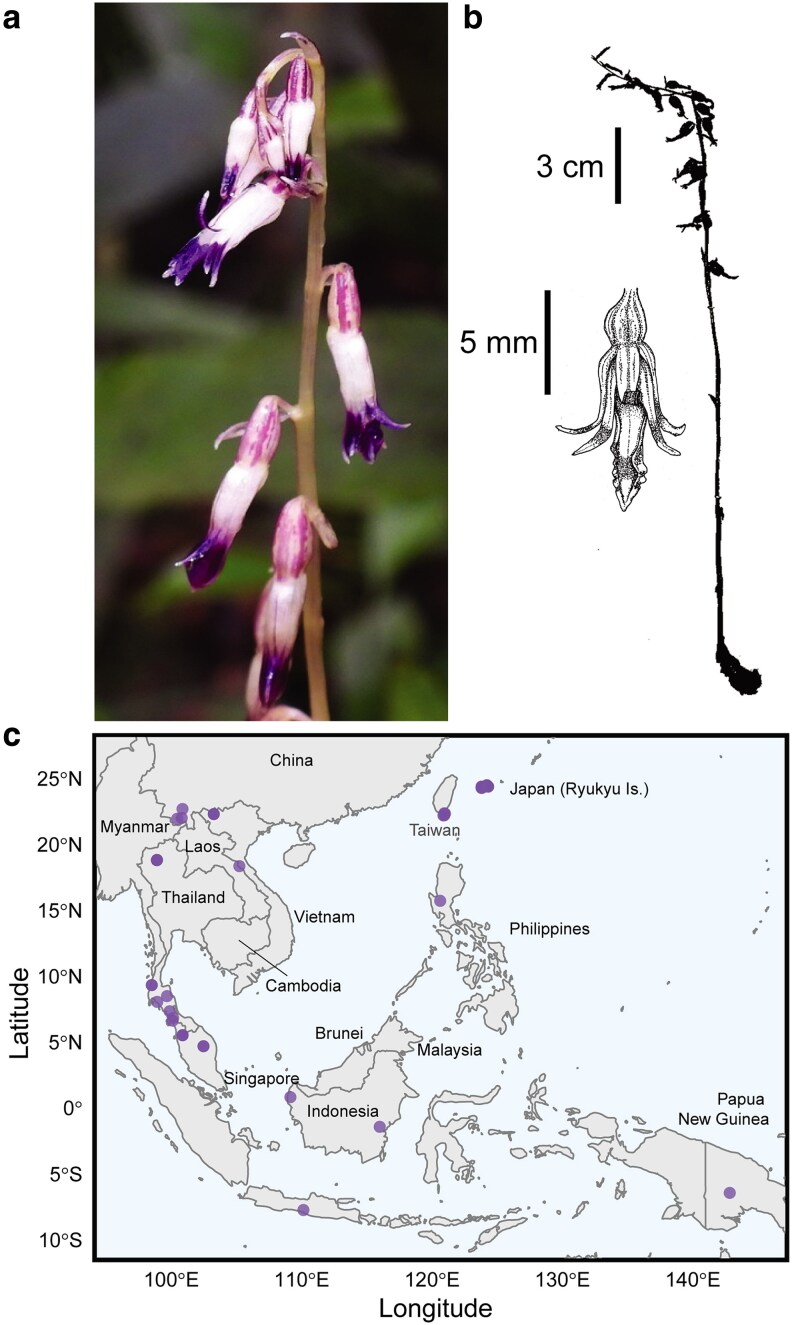
a) Inflorescence of *Stereosandra javanica* (photo credit: Chen Shu, 12 June 2022, https://www.inaturalist.org/observations/121542719, image licenced under CC BY-NC 4.0, accessed 03 June 2025). b) Habit and floral illustration of *Stereosandra javanica* (illustration: C. Barrett). c) Map of occurrence data (‘preserved specimen’) downloaded from the Global Biodiversity Information Facility (https://doi.org/10.15468/dl.y8tg3v, accessed 03 June 2025).

Tribe Nervilieae is composed of three genera: the leafy *Nervilia* (83 accepted species), and two leafless genera, *Epipogium* (eight accepted species) and the monotypic *Stereosandra*, represented by *S. javanica* Blume (Pridgeon et al. 2009, Freudenstein and Barrett 2010, Merckx et al. 2013a, Chase et al. 2015, POWO 2025). While *Nervilia* and *Epipogium* have been more widely studied from phylogenetic and genome evolutionary perspectives (Schelkunov et al. 2015, Minasiewicz et al. 2022, Zhao et al. 2024, Gale et al. 2025), *Stereosandra javanica*: (i) has been sparsely collected (89 recorded collections; https://www.gbif.org; accessed 07 June 2025), (ii) has not been included in any published phylogenetic study to date, and (iii) has no publicly available genetic data (i.e. zero records in NCBI GenBank; accessed 07 June 2025). *Stereosandra javanica* is geographically widespread but rare throughout southeastern Asia from southern China to Papua New Guinea and the Ryukyu Islands of Japan (Fig. 1). This species superficially resembles the related, fully mycoheterotrophic *Epipogium* but has relatively reduced flowers that are likely adapted to self-pollination (Fig. 1; Holttum 1953; Seidenfaden and Wood 1992).

Within Nervilieae, the plastomes of *Epipogium* are known to be among the most drastically reduced in plants: *E*. *aphyllum* Sw. has a plastome of just over 30 kb, with 26 genes, and *E. roseum* (D. Don) Lindl. has a plastome of 19 kb and 28 genes, with the shorter length of the latter attributed to the loss of the Inverted Repeat (Schelkunov et al. 2015). This is in stark contrast to what is known in the leafy *Nervilia*, with plastomes > 160 kb and a full set of plastid genes based on the few species for which plastid genomes have been sequenced (Wen et al. 2022). Is *Stereosandra* sister to *Epipogium*, representing a single putative transition to full mycoheterotrophy within Nervilieae, or does it represent an independent trajectory in the tribe? We sequenced the first plastid genome of *Stereosandra javanica* and further investigated its phylogenetic position using nuclear sequence capture to address the history of mycoheterotrophy in Nervilieae. Our objectives were to (i) characterize plastid genome structure in *Stereosandra*, (ii) resolve and provide support for its phylogenetic placement, (iii) explore evidence for relaxed selection on plastid genes in *Stereosandra*, and (iv) infer the number of independent transitions to full mycoheterotrophy in Nervilieae.

## Materials and methods

### Sampling, sequencing, and data processing

Tissues were collected from five representatives of Nervilieae: four from herbarium specimens and one from a field collection (Table 1). Approximately 0.2 g of herbarium or silica-dried leaf material was used for DNA extraction with the CTAB method, with an additional 24:1 v:v chloroform:isoamyl alcohol precipitation step (Doyle and Doyle 1987). DNA quality and quantity were assessed with 1% agarose gels and the Qubit Broad Range dsDNA Assay, respectively (Thermo Fisher, Waltham, Massachusetts, USA). The SPARQ DNA Frag and Library Kit (Quantabio, Beverly, Massachusetts, USA) was used to build Illumina libraries at 0.4× volumes. Libraries were quantified with Qubit High Sensitivity dsDNA Assay, and each library was split in half, with one reserved for genomic DNA sequencing (genome skimming) and the other for nuclear sequence capture. Pooling, DNA quantification, fragment size analysis, sequence capture with the Angiosperms353 kit (Johnson et al. 2019), and sequencing were conducted as in (Barrett et al. 2024, 2025). Briefly, sequence capture and genome skimming libraries were sequenced on separate runs of an Illumina NextSeq2000 (Illumina Inc., San Diego, California, USA), with a P2 300-cycle run for sequence capture and a P3 200-cycle run for genome skimming. Libraries were sequenced with 61 and 54 other samples, respectively, from other projects.

**Table 1:** Specimens used in this study.

Species	Source	Code	Collection	Year	Locality
*Nervilia concolor* (Blume) Schltr. [ = *Nervilia aragoana* Gaudich., syn. *Pogonia concolor* (Blume) Blume] **(T)**	herbarium	US84	Fosberg 64536 (US)	1985	Guam, USA
*Epipogium aphyllum* Sw. **(T)**	herbarium	NY38	Coste 1431 (NY)	1913	France
*Stereosandra javanica* Blume **(T)**	herbarium	US31	Fosberg 37285 (US)	1956	Okinawa, Japan
*Stereosandra javanica* Blume **(T)**	herbarium	US32	Ramos 12124 (US)	1910	Luzon, Philippines
*Stereosandra javanica* Blume **(T)**	field	OS2	Mennes 10 (L)	2013	Malaysia

(T) = type species of the genus. Herbarium codes (under ‘Collection’): L = Naturalis Biodiversity Center, Leiden, the Netherlands; NY = New York Botanical Garden Sterre Herbarium, New York, New York, USA; US = Smithsonian National Museum of Natural History, Washington, DC, USA. ‘Code’ = unique designation used in downstream analyses, and ‘Year’ = year the specimen was originally collected.

FASTQ files for each individual accession were merged between runs using a BASH script, and read files for additional orchid taxa generated by Angiosperms353 capture or RNA-seq were downloaded with SRA-TOOLS v3.2.1 (https://github.com/ncbi/sra-tools). Downloaded files were renamed using a custom python script (https://github.com/barrettlab/2025_Nervilieae). FASTP v0.23.4 (Chen et al. 2018) was used to remove adapters, quality-trim, and filter reads (*-w 16 –trim_poly_g –trim_poly_x -l 35 –cut_right*).

### Plastid genome analyses

Cleaned reads were used to attempt complete plastid genome assemblies for *Stereosandra* with GetOrganelle v1.7.7.1 (Jin et al. 2020), using kmer lengths (21, 31, 45, 55, 65, 75, and 85), 100 rounds of contig extension using all reads, and *Nervilia fordii* (Hance) Schltr. (NCBI GenBank accession number ON515491) as the reference genome. The draft genome was verified by mapping reads to the genome in Geneious v10.2.6 (https://www.geneious.com). Inverted repeat boundaries were identified using the Geneious repeat finder and verified with paired-end reads. The genome sequence was annotated with GESEQ (Tillich et al. 2017) using the same *Nervilia* accession, and the GenBank flat file was edited in Geneious to verify reading frames using the open reading frame finder and the *Nervilia* annotation. Pseudogenes were annotated based on internal stop codons or truncations of >25% in length relative to the genes from *Nervilia*. The GenBank file was converted to a feature table with GB2SEQUIN (Lehwark and Greiner 2019).

An alternative approach was taken for two *Stereosandra* herbarium accessions (Table 1), as assemblies with GetOrganelle consistently failed, likely due to similar plastid and mitochondrial coverage depths, resulting in contigs of unknown affinity based on BLAST searches. Cleaned reads were mapped to the reference plastome with BWA-MEM v2, which supports gapped read alignment, preserving information on insertions and deletions (Vasimuddin et al. 2019). BCFTOOLS v1.17 was then used to call haploid variants and build a fasta consensus among the reads (Danecek et al. 2021). The resulting BAM and FASTA consensus files were then imported into Geneious to verify the contiguity and coverage of the assemblies. The finished assemblies were annotated as above.

Representative plastome annotations were downloaded from GenBank, to which the *Stereosandra* annotation for OS2 was added (Table 1). Whole plastomes were aligned with the progressiveMAUVE plugin for Geneious with the second copy of the inverted repeat removed (where applicable) to detect plastome structural inversions (Darling et al. 2010). To visualize gene presence and absence among members of Nervilieae, we used a custom R script (https://github.com/barrettlab/2025_Nervilieae) that takes a multi-GenBank flat file as input, splits this into individual files, builds a data frame of gene presence with standardized gene names, sorts taxa on plastome length, and plots genes as present or absent.

### Phylogenomic analyses of plastid and nuclear DNA

Protein coding DNA sequences (CDS) were extracted with PhyloSuite v1.2.3 (Zhang et al. 2020). Each CDS was aligned with MAFFT v7.525 (Katoh and Standley 2013) and then realigned with the codon-aware MACSE v2.0.7 (‘refineAlignment’ option with all other parameters as default; Ranwez et al. 2018). Phylogenetic analyses based on the concatenated CDS matrix were conducted with IQTree2 v2.4.0 (Minh et al. 2020) using MixtureFinder (Ren et al. 2025) to identify the best-fit mixture model configuration. MixtureFinder has several advantages in that it does not rely on *a priori-*specified partitioning and deals with rate heterogeneity and heterotachy, or lineage-specific rate variation across branches and over time (Philippe et al. 2005, Ren et al. 2025). Nervilieae is composed of both leafy-autotrophic (*Nervilia*) and leafless-heterotrophic taxa (*Epipogium*, *Stereosandra*), and *Epipogium* was shown previously to exhibit accelerated plastid substitution rates and missing data due to genomic deletions (Schelkunov et al. 2015, Zhao et al. 2024). Two analyses were run, one including and one excluding *Epipogium*. Branch support was assessed with 1000 ultrafast bootstrap replicates (Hoang et al. 2018). In addition, plastomes of *Stereosandra* were aligned with *Nervilia fordii* as an outgroup taxon with one Inverted Repeat copy removed in MAFFT.

Newly generated and SRA-downloaded data were processed with HybPiper v2.3.2, using the ‘newtargets’ Angiosperms353 reference loci, which were shown to improve the number and length of loci assembled over the original set (Johnson et al. 2016, McLay et al. 2021). Assembled loci representing the ‘best paralog’ set were aligned with MAFFT and imported into Geneious. Loci with fewer than five representative accessions were removed. IQTree2 was used to build individual gene trees and to analyse the concatenated matrix with 1000 ultrafast bootstrap replicates. MixtureFinder was used to select the best overall mixture configuration for the concatenated data and for each individual CDS alignment, the latter using a BASH script (https://github.com/barrettlab/2025_Nervilieae). Weighted ASTRAL (wASTRAL v1.19.3.7) was used to infer a coalescent species tree using the hybrid method which accounts for gene tree uncertainty by incorporating branch length and clade support information from the gene tree input (Mirarab et al. 2014, Zhang and Mirarab 2022).

### Analysis of selection

RELAX in HyPhy v2.5.73 was used to test evidence for relaxed or intensified negative selection in *Stereosandra* for each plastid CDS alignment, using the plastid topology (Pond et al. 2005, Wertheim et al. 2015, Kosakovsky Pond et al. 2020). *Epipogium* was excluded from the analyses due to having few genes present as functional copies, and to remove the potential effects of increased substitution rates among the reference branches, with *Stereosandra* alone being specified as the test branch and all other leafy orchids as reference branches. Only CDS for which *Stereosandra* had a non-pseudogenized copy were analysed. The HyPhy-RELAX output was summarized with a BASH script to create a data frame for plotting with an R script (https://github.com/barrettlab/2025_Nervilieae). Secondary structure predictions for MatK in *Stereosandra* were conducted with the JPred4 server (https://www.compbio.dundee.ac.uk/jpred/faq.shtml; Drozdetskiy et al. 2015), which uses neural networks to classify amino acids as belonging to α-helix, β-strand and coil, or other secondary structures. The predicted amino acid sequences for MatK were extracted from PhyloSuite and an R script was used to plot unique amino acid replacements for all accessions (i.e. those not shared with any others), along with the secondary structural predictions. The ‘Tether’ and ‘Domain X’ regions of MatK were also mapped to the alignment and plotted in R.

### Ancestral state reconstruction

We used stochastic character mapping to infer the history of trophic mode among representatives of Nervilieae and orchid outgroup taxa in PHYTOOLS v2.0 (Huelsenbeck et al. 2003, Revell 2012, 2024). We used the nuclear coalescent (species) tree, with branch lengths converted to substitutions per site in CASTLES v1.3.0 (Tabatabaee et al. 2023). Because orchids have a notoriously poor fossil record (Ramírez et al. 2007, Conran et al. 2009, Poinar and Rasmussen 2017), we were unable to generate a fossil-calibrated chronogram for ancestral state reconstruction. Instead, we used the ‘chronos’ function in the R package APE (Paradis 2013, Paradis and Schliep 2019), which uses penalized likelihood rate smoothing, to generate an ultrametric tree. Trophic mode was specified as a binary state matrix (0/1) in which reversals from leafless/fully mycoheterotrophic to leafy/autotrophic were restricted. We used 1000 mappings, summarized as pie charts and posterior probabilities on the tree.

## Results

### Plastid genome structure and content in *Stereosandra*

**Figure 2:**
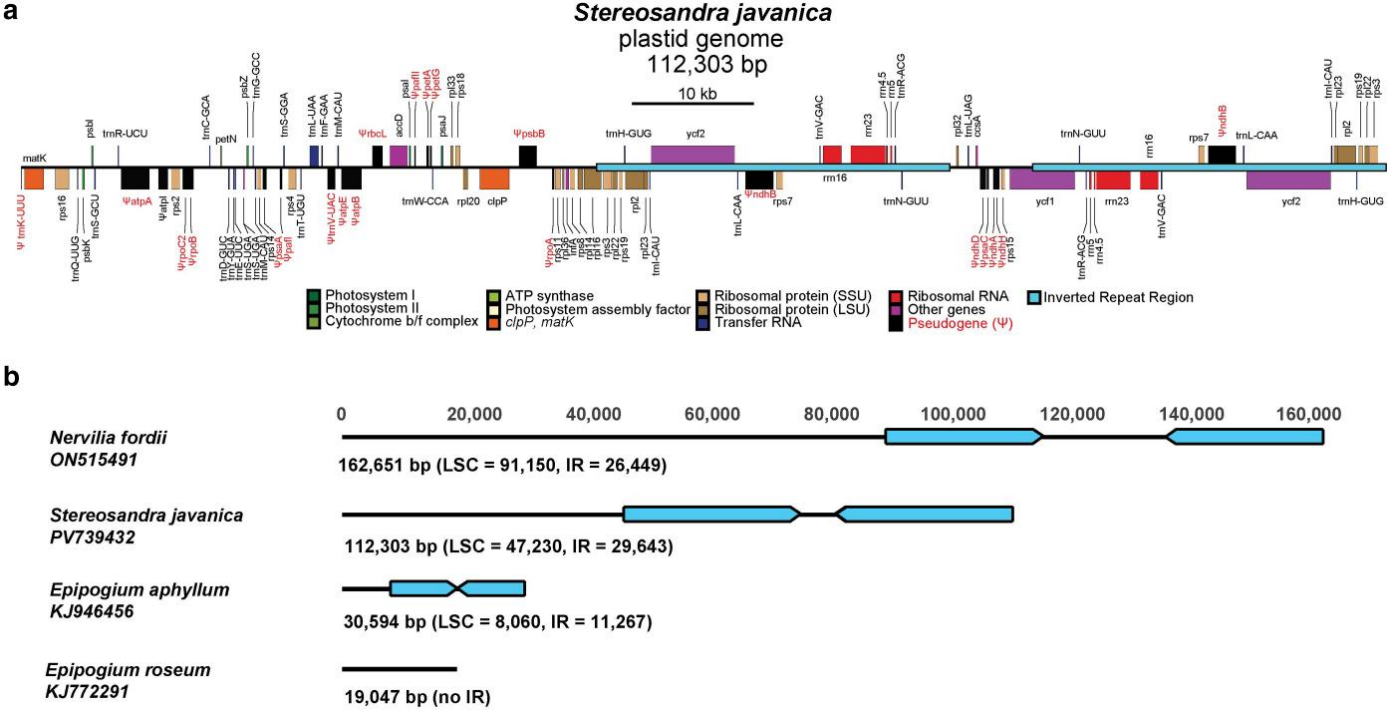
a) Plastome map of *Stereosandra javanica*. Scale bar = 10 kilobases. Genes are coloured according to functional classes, with pseudogenes (Ψ) as black rectangles with red font. b) Overview of plastome lengths among the three genera of Nervilieae. Scale is in base pairs. Numbers below each plastome indicate the total plastome length (bp), and the numbers in parentheses are the lengths of the Large Single Copy Region (LSC) and Inverted Repeat (IR) in base pairs. The Inverted Repeats, if present, are coloured in light blue.

*De novo* plastome assembly for the three *Stereosandra* accessions with GetOrganelle was successful in one instance (accession OS2) sourced from the field, but not for the two accessions sourced from herbarium material (US31 and US32; Table 1). The OS2 plastome was 112 303 bp in length (Table 2; Fig. 2). The Large Single Copy region (LSC) was 47 230 bp, the Small Single copy was 5787 bp, and the Inverted Repeat (IR) was 29 643 bp. Relative to *Nervilia fordii*, the IR of *Stereosandra* is expanded by 1688 bp at the LSC-IR_B_ boundary to include *rps3* and the 5′ exon of *rpl16*, and by 1506 bp at the IR_B_-SSC boundary to include a large portion of *ycf1* (Fig. 2). The GetOrganelle assembly for accession US31 resulted in two scaffolds. The first was 2372 bp and corresponded via BLAST searches against the OS2 plastome to a region of the IR that included the four plastid ribosomal RNA genes. The second scaffold was 726 624 bp but had no significant BLAST hits. The US32 assembly had six scaffolds from 1208 to 111 052 bp. Only the 1208 bp scaffold had a positive BLAST hit, to plastid *rps19* and *trnH-GUG* (both 100% identity). Stringent mapping and variant calling with BWA-MEM and BCFTOOLS, however, recovered complete plastomes for both accessions (Table 2). Assembled plastome lengths and coverage depths were 112 209 bp and 23.6× (US31), 112 267 bp and 21.9× (OS2; Table 2), representing a 31% reduction in total plastome size relative to the leafy *Nervilia fordii*.

**Table 2:** Plastome assembly summary for *Stereosandra javanica* accessions.

Code	Collection	Reads	Mapped	Depth	% plastid	Length	GenBank
OS2	Mennes 10 (L)	30 015 348	381 472	249.4	1.27	112 303	PV739432
US31	Fosberg 37285 (US)	36 562 369	53 352	23.6	0.14	112 209	PV763472
US32	Ramos 12124 (US)	46 970 140	41 864	21.9	0.08	112 267	PV763473

Herbarium codes (under ‘Collection’): L = Naturalis Biodiversity Center, Leiden, the Netherlands; NY = New York Botanical Garden Steere Herbarium, New York, New York, USA; US = Smithsonian National Museum of Natural History, Washington, DC, USA. ‘Reads’ = total number of read pairs post-filtering, ‘Mapped’ = total number of reads mapped to Stereosandra javanica OS2, ‘Depth’ = mean coverage depth of the plastome (× coverage), ‘% Plastid’ = the percentage of plastid reads among the total genomic reads, ‘Length’ = plastome length in bp, and ‘GenBank’ = NCBI GenBank accession number.

**Figure 3:**
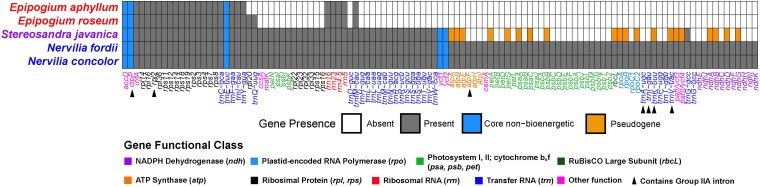
Presence or absence of putatively functional genes among representatives of tribe nervilieae. Grey = present and putatively functional with an open in-tact Reading frame, white is absent (deleted from the genome) or presumably nonfunctional (pseudogene), and light blue indicates the five ‘core non-bioenergetic’ genes that are hypothesized to be among the last to be lost in heterotrophic plants (*sensu* Graham et al. 2017). Gene names are coloured by functional class. Black arrowheads indicate genes containing Group IIA introns.

Based on the *Stereosandra* OS2 annotation, there were 32 putatively functional CDS, 23 tRNA genes, and four rRNA genes. This represents a 49% reduction in functional gene content relative to *Nervilia* (with 113 functional genes; Fig. 3). By comparison, *Epipogium* represents 77% (*E. aphyllum*) and 75% reductions (*E. roseum*) relative to *Nervilia*. *Epipogium* had no pseudogenes, whereas *Stereosandra* had evidence for 20 (Fig. 3). Among these were: *ndhA, B, D*, and *G* (NADPH Dehydrogenase complex); *rpoA*, *B*, and *C2* (Plastid-Encoded RNA Polymerase); *petA* and *G* (Cytochrome b_6_/f complex); *psaA*, *C*, and *psbB* (Photosystems I and II); *rbcL* (RuBisCO Large Subunit); *atpA*, *B*, and *E* (ATP Synthase); *pafI*/*ycf3* and *pafII*/*ycf4* (Photosystem Assembly/Stability proteins); and *trnK*-*UUU* and *trnV*-*UAC* (Transfer RNAs; Fig. 3).

### Phylogenomic analyses of plastid and nuclear DNA

**Figure 4:**
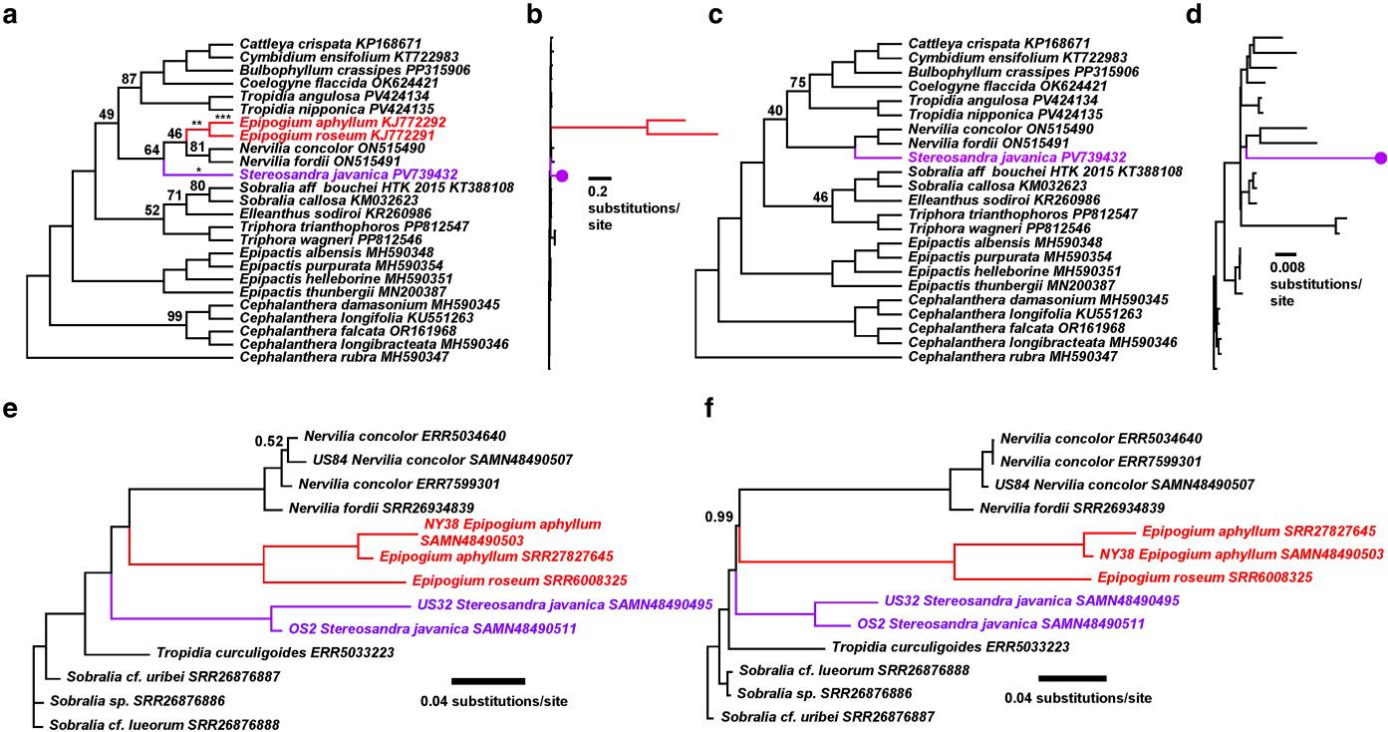
Phylogenetic relationships inferred among members of the Nervilieae and outgroup taxa. a) Cladogram of inferred plastid relationships based on 83 concatenated plastid genes under the best-fit mixture model configuration (see Table 3 for model details). ‘*’ indicates the 47 independent gene losses in the branch leading to *Stereosandra* (in Fig. 3), ‘**’ indicates the shared gene losses in *Epipogium aphyllum* and *E. roseum*, and ‘***’ indicates the two additional gene losses in *E. aphyllum*. b) Phylogram showing branch length estimates, with the two fully mycoheterotrophic *Epipogium* species in red and the fully mycoheterotrophic *Stereosandra* in purple (purple circle). c) Cladogram of inferred plastid relationships with *Epipogium* removed. d) Phylogram showing branch lengths with *Epipogium* removed, as before. e) Phylogram showing inferred relationships of 295 concatenated nuclear loci (Angiosperms353). f) Phylogram showing inferred relationships based on wASTRAL coalescent analysis of 295 gene trees, with branch lengths converted to substitutions per site with CASTLES. All scale bars = substitutions per site. Bootstrap support is shown in a), c), and e); local posterior probabilities (LPP) are shown in f) above each branch. Blank values above branches indicate Bootstrap = 100% or LPP = 1.0.

The tree inferred for plastid DNA under the best-fit mixture model (Table 3) revealed weak support for relationships among genera within Nervilieae when including *Epipogium* (Fig. 4a). The two *Epipogium* species were inferred as sister to one another (Bootstrap Support, or BS = 100), and together sister to *Nervilia* but with no support (BS = 46). *Stereosandra* was inferred as sister to the rest of *Nervilieae* but with weak support (BS = 64), and Nervilieae were inferred as sister to a clade of Tropidieae + representatives of the Core Epidendroideae (CE) but with no support (BS = 49). Plastid analysis with the removal of *Epipogium* resulted in a strongly supported sister relationship between *Stereosandra* and *Nervilia* (BS = 100), but Nervilieae had no support for its inferred sister relationship to (Tropidieae, CE) (BS = 40; Fig. 4b). Inferred nuclear DNA relationships within Nervilieae were strongly supported in both the concatenated and coalescent (wASTRAL) analyses (Fig. 4c and d). In both trees, relationships of (*Stereosandra*, (*Epipogium*, *Nervilia*)) were recovered, with all nodes supported with BS = 100 in the concatenated tree and Local Posterior Probabilities (LPP) > 0.99 in the coalescent tree. Relative differences in inferred branch lengths were noticeably more pronounced for *Epipogium* in the plastid tree (Fig. 4a) when compared with the nuclear trees (Fig. 4c and d). Analysis of gene tree concordance and discordance revealed 80 gene trees concordant with the inferred WASTRAL species tree vs. five discordant for the monophyly of Nervilieae (Supplementary Figure S1). We recovered 51 concordant and 24 discordant resolutions for the sister relationship of *Stereosandra* and (*Epipogium*, *Nervilia*) and 80 concordant vs. 106 discordant resolutions for (*Epipogium*, *Nervilia*).

**Table 3:** Summary of alignments and phylogenomic analyses.

	Plastome concatenated	Plastome concatenated (no *Epipogium*)	Nuclear A353 (coalescent)	Nuclear A353 (concatenated)
Alignment L	70 560	70 328	295 gene trees	268 481
Taxa	25	23	14	14
PIC	5745	4767	14 502	14 502
Missing	0.1491	0.0826	*16%	0.4754
Model	{TVM + FO,K81u + FO,TIM + FO,TVM + FO,F81 + FO,GTR + FO,F81 + FO,F81 + FO}+R3	{TVM + FO,K81u + FO,TVM + FO,TPM2 + FO}+R3	n/a	{TIM2 + FO,TPM2u + FO,F81 + FO,TIM + FO,TIM + FO,JC + FO,TVM + FO}+G
NP	100	71	6843	n/a
lnL	−183 988	−172 311	−594 989	−610 375
BIC	369 091.6	345 413.9	1 275 520	1 221 538
wBIC	1	1	n/a	0.535

‘Alignment L’ = total alignment length in bp, ‘Taxa’ = the number of taxon accessions included, ‘PIC’ = the number of parsimony informative characters, ‘Missing’ = the percentage of missing data (with * indicating the total percentage of taxa missing from all individual gene alignments), ‘Model’ = the best fit mixture model configuration (with ‘n/a’ for the coalescent analysis due to different mixture model configurations being determined for each individual alignment), ‘NP’ = the number of free parameters in the model (or the ensemble of mixture models in the coalescent analysis), ‘lnL’ = the log-likelihood of the data, ‘BIC’ = the Bayesian Information Criterion score of the model, and ‘wBIC’ = BIC weight of the model compared to all alternatives tested.

### Analysis of selection

**Figure 5:**
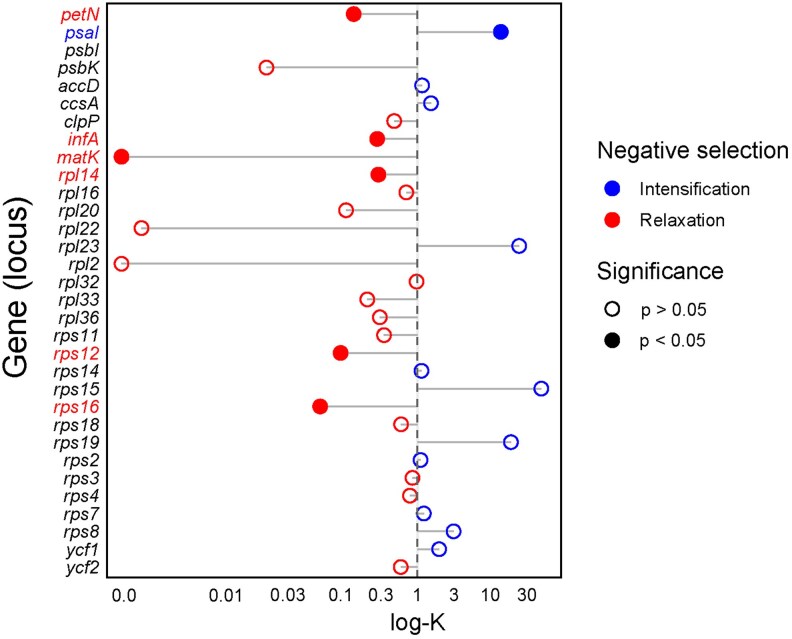
Results of RELAX analysis in HyPhy showing inference of relaxed (red, left) or intensified (blue, right) negative selection, treating *Stereosandra* as the test branch. Filled circles indicate significant relaxation or intensification of negative selection (*P* < .05). Only genes retained as putatively functional in *Stereosandra* were included. Each gene is listed along the *y*-axis, while the log of the RELAX intensification/relaxation parameter (k) is on the *x*-axis.

Inference of selection on individual genes, treating *Stereosandra* as the test branch and removing *Epipogium*, revealed significantly intensified selection in one gene (*psaI*), and significantly relaxed selection for six genes (*infA, matK, petN, rpl14, rps12*, and *rps16*; Fig. 5). Among the genes displaying evidence of relaxed selection, one is a photosynthesis-related gene (*petN*), four function in translation (*infA, rpl14, rps12*, and *rps16*), and one functions in removal of Group IIA introns (*matK*). Notably, *Stereosandra* retains only three genes with Group IIA introns: *clpP* (intron 2), *rpl2*, and *rps12* (3′ intron); *Epipogium* also retains these genes and their introns but has lost *matK* (Fig. 3). A summary of mycoheterotrophic orchid plastomes which have lost or retained *matK*, and the status of their corresponding group IIA intron-containing genes is shown in Supplementary Figure S2. Several taxa retain *matK* but have lost various Group IIA intron-containing genes, and several of them have the same configuration observed in *Stereosandra*.

*Stereosandra* has over twice the number of unique amino acid changes than any other taxon, i.e. 56 amino acid replacements in *Stereosandra* are not shared with any other taxa out of 512 positions in the predicted MatK protein (Supplementary Figure S3). Many replacements in *Stereosandra* occur in α-helix or β-sheet motifs, and further, some are located within the ‘Tether’ and ‘Domain X’ regions, which are both near the C-terminus of the MatK protein product (Supplementary Figure S3). While *accD* was not shown to be under relaxed selection in *Stereosandra* (Fig. 5), we detected a modification at the 3′ end of the gene when comparing the three sequenced accessions (Supplementary Figure S4). This involves an insertion or deletion of 7 bp between OS2 and US31/US32. Furthermore, the indel motif is a repeat (TTGAATA), with two copies in OS2 and one copy in US31 and US32, causing an earlier stop codon in the former or a later stop codon in the latter.

### Ancestral state reconstruction

**Figure 6:**
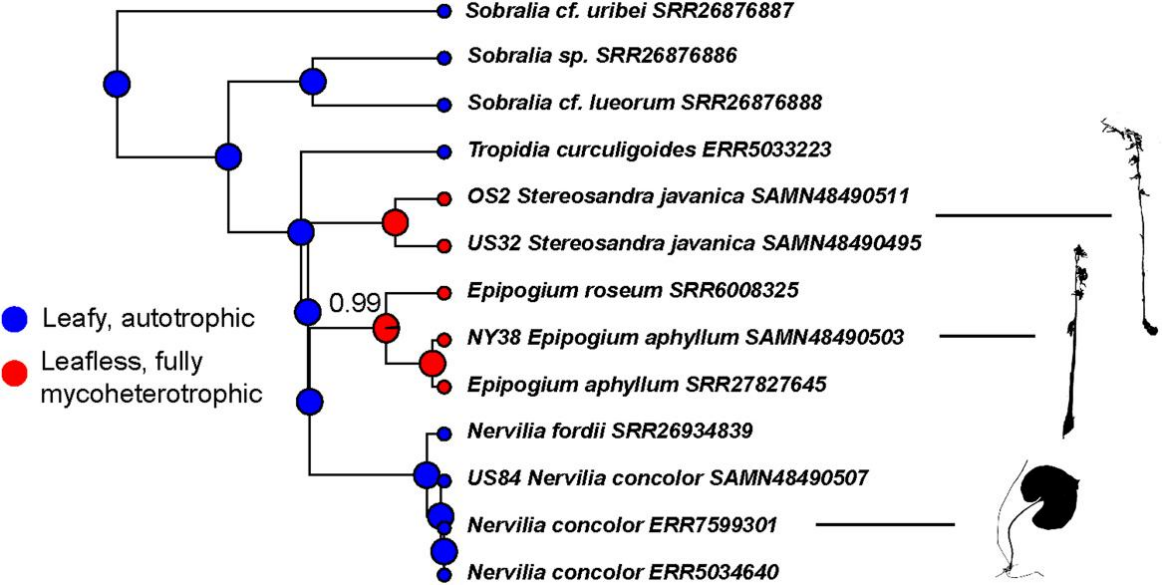
Ancestral state reconstruction of leafiness/trophic mode via stochastic character mapping. Pie charts summarize 1000 mappings with blue = leafy, autotrophic and red = leafless, fully mycoheterotrophic. Numbers at nodes indicate the posterior probability of the most probable state; blank nodes indicate posterior probability = 1.0. Silhouettes: *Stereosandra javanica* (top), *Epipogium aphyllum* (middle), and *Nervilia concolor* (= *N. aragoana*, bottom). Silhouettes were drawn from herbarium specimen images in the Southeastern Regional Network of Expertise and Collections, for *Epipogium aphyllum* [collector s.n., specimen barcode CHRB0097469 (CHRB)] and *Nervilia concolor* [Raulerson 13856, NLU0383355 (USCH)]. The image for *Stereosandra javanica* was made from [Kerr 306, K000595882 (K)]. Images are licenced under http://creativecommons.org/licenses/by/3.0 and http://creativecommons.org/licenses/by/1.0. Herbarium codes: CHRB (The Chrysler Herbarium, Rutgers University, USA), K (Kew Herbarium, UK), and USCH (A.C. Moore Herbarium, University of South Carolina, USA).

Stochastic character mapping under the no-reversal model inferred a leafy, autotrophic ancestor for Nervilieae (posterior probability, or PP = 1), and for the inferred common ancestor of (*Epipogium*, *Nervilia*; PP = 1) (Fig. 6). Thus, we provide evidence for two independent transitions to losses of leaf laminae and (presumably) photosynthesis in the tribe, based on the relationships inferred from the nuclear coalescent analysis (Fig. 4d) and the distribution of character states in the terminal taxa.

## Discussion

### Plastome structure and content in *Stereosandra*

Nervilieae, with just three genera, spans the spectrum of reductive plastid genome evolution with: the leafy, autotrophic *Nervilia* (∼160 kb) representing a full suite of functional genes; *Epipogium* representing severe plastome reduction (∼19 and 30 kb), retaining only 26–28 genes with no detectable pseudogenes; and *Stereosandra* representing a ‘transitional’ stage in plastome degradation (∼112 kb) with rampant gene loss and evidence of pseudogenes. Overall plastome length may be misleading in the case of *Stereosandra*, as there has been a nearly 3 kb expansion of the IR relative to *Nervilia*, but a 48% reduction of the LSC region, which harbours most photosynthesis-related genes (Fig. 2). Considering proposed conceptual ‘models’ of plastome degradation, *Stereosandra* could be placed in ‘Stage 4-5’ of the models of Barrett and Davis (2012) and Barrett et al. (2014), which include losses of the *ndh* complex, photosynthesis-related genes, the *rpo* complex, the *atp* complex, and some housekeeping genes (tRNA genes). *Stereosandra* corresponds to ‘Stage 3’ in the model of Wicke et al. (2016), having lost ‘…genes that have a prolonged function (e.g. *atp* genes, *rbcL*) and nonessential housekeeping genes’. This would also fit the less stringently defined ‘Loss of the bulk of photosynthesis genes’ *sensu* Graham et al. (2017).

Although we did not conduct formal divergence time analysis due to previously mentioned limitations, the order of divergence in Nervilieae is informative on the relative timing of plastome degradation. If our inferred nuclear topology reflects reality, then genomic losses would have been more rapid in *Epipogium* than *Stereosandra*, given the earlier divergence of *Stereosandra*. Pérez-Escobar et al. (2024) inferred the stem age for *Nervilia* (the only representative of Nervilieae in that study) at ca. 50 Ma. Zhang et al. (2023) included one species of *Nervilia* and two species of *Epipogium* and inferred a stem age for the Nervilieae 59–65 Ma, and a crown age for (*Epipogium*, *Nervilia*) between 52 and 58 Ma, but *Stereosandra* was not included. The short time between the inferred crown and stem ages of Nervilieae from Zhang et al. (2023) means that *Stereosandra* would have split off within this narrow window, and like many other divergences among lineages of the EDE, these would have occurred rapidly. If previous inferences reflect reality, then plastomes of *Epipogium* would have taken at most up to 58 million years to reach their current states, while *Sterosandra* would have had more time as a distinct lineage, but in fact *Stereosandra* shows less evidence of degradation. While it may be tempting to draw conclusions on the rate of degradation over time, we refrain from doing so given the multiple levels of uncertainty in orchid divergence time analyses, and importantly, the possibility of extinct leafy ancestors in each of the lineages that comprise the extant genera of Nervilieae. In other words, we can’t say *when* the transition to full mycoheterotrophy occurred within each genus.

One component of proposed ‘models’ of plastome degradation currently lacking is the presence of pseudogenes. The hypothesis is that as the transition to full mycoheterotrophy (or holoparasitism for that matter) proceeds, relaxed selective pressures on photosynthesis manifest as loss-of-function mutations with negligible fitness consequences, resulting in pseudogenes. Eventually these are lost due to deletions in the plastome; in a sense, purged from the genome (functional gene-pseudogene-deletion). However, in mycoheterotrophic plastomes that are highly reduced, pseudogenes are rarely if ever observed (e.g. *Epipogium*, *Gastrodia*, *Pogoniopsis* among the orchids). Presumably, regions that ancestrally harboured pseudogenes have been deleted, analogous to a ratcheting effect in which plastomes unidirectionally shrink over time.

While models have focused on which functional genes are *retained* in late-stage heterotrophs, detectable pseudogenes may be an overlooked yet important ‘parameter’ in these models and their absence may be a hallmark of minimal plastomes. Whether eventual deletion of pseudogenes is a neutral process (deletions with no fitness consequences due to relaxed negative selection) or an example of positive selection for smaller plastomes (i.e. fitness benefits of having to replicate and maintain smaller plastid genomes) is up for debate (Barrett and Disbrow, *in review*). It is informative to compare heterotrophic plastid genome evolution to that in other genomic compartments: while minimal mitochondrial genomes have been reported in some parasites (e.g. as small as 42 kb in the hemiparasitic *Viscum* L.; Skippington et al. 2015), in other cases they are expanded (>1 Mb in some holoparasitic Orobanchaceae e.g. Kim et al. 2025). In the case of the fully mycoheterotrophic orchid *Gastrodia*, the mitochondrial genome comprises dozens of circular structures, exceeding 2 Mb in *G. javanica* Endl. (e.g. Wang et al. 2025), though mitochondrial genome sequencing lags behind plastid genome sequencing, with only a handful of genera having complete mitogenomes available. If plastid genome degradation in heterotrophic plants is a neutral process resulting from relaxed selective pressures, then it would be just as reasonable to expect plastid genomic expansion as it would compaction. As far as we know, there are no documented cases of plastid genome expansion in association with the loss of photosynthesis.

### Phylogenomic analyses of plastid and nuclear DNA

Our findings of weak plastid and strong nuclear support for placement of genera at the base of the epidendroid orchids match emerging trends from recent phylogenomic studies (Serna-Sánchez et al. 2021, Wong and Peakall 2022, Zhang et al. 2023, Barrett et al. 2024, Pérez-Escobar et al. 2024). We conclude that accelerated plastid substitution rates and high amounts of missing plastid data in fully mycoheterotrophic lineages, concentrated among the EDE tribes (in addition to previously inferred rapid divergence times), are the primary reasons for phylogenetic uncertainty in this region of the orchid tree. Most recently, Serna-Sánchez et al. (2021) conducted a family-wide plastome analysis of the Orchidaceae, including representatives from the EDE. While many relationships were supported, others were not, e.g. a clade of (*Triphora*, (*Nervilia*, *Tropidia*)), but few mycoheterotrophic taxa were included in the study and only *Nervilia* from Nervilieae was included. Klimpert et al. (2022) analysed orchid relationships with emphasis on the mycoheterotrophic *Pogoniopsis schenckii* Cogn., which has the most reduced plastome known among the orchids at ∼14 kb. The inclusion of *Pogoniopsis* in their plastid analysis led to severe distortion of relationships and support due to long branch attraction, with *Pogoniopsis*—hypothesized to be a member of the EDE tribe Triphoreae—grouping with strong support as nested within the CE, sister to members of the tribe Vandeae Lindl. Their analysis of mitochondrial DNA, however, placed *Pogoniopsis* among the EDE tribes, sister to *Sobralia* Ruiz & Pav., although no other members of Triphoreae were included in the mitochondrial analysis. Their interpretation was that the lower substitution rates in the mitochondrial genome may mitigate impacts of long branch attraction observed with plastid data. Again, only *Nervilia* was included from Nervilieae.

Barrett et al. (2024) sampled representatives of all eight EDE tribes, focusing on the mycoheterotrophic *Wullschlaegelia*. Based on a nearly complete set of plastid genes, and models explicitly accounting for heterotachy (GHOST heterotachy model; Crotty et al. 2020), they recovered a strongly supported ‘long branch’ clade composed exclusively of mycoheterotrophic taxa: *Wullschlaegelia* (Wullschlaegelieae), *Gastrodia* and *Didymoplexis* (Gastrodieae), *Pogoniopsis* (Triphoreae), and *Epipogium* (Nervilieae). However, using nearly full mitochondrial gene sets, they still recovered *Wullschlaegelia* within a clade of *Pogoniopsis* and *Triphora* (Triphoreae) with moderate to weak support. They concluded that even though models have explicitly been developed to deal with rate heterogeneity, there may be no way to ‘model one’s way out’ of extreme cases of long branch attraction (and missing data) in plastid-based analyses of heterotrophs, and that organellar DNA, especially plastid DNA, may be uninformative, and perhaps positively misleading in cases such as the mycoheterotroph-rich EDE.

Most recently, nuclear phylogenomic datasets and coalescent analysis have been used to resolve relationships in orchids and have included at least some representative sampling of the EDE, including mycoheterotrophs. Pérez-Escobar et al. (2024) used Angiosperms353 loci and coalescent analysis to resolve relationships across the orchids and included representatives of seven of the eight EDE tribes. However, only *Nervilia* from Nervilieae was included in their study. Zhang et al. (2023) used transcriptome sequencing and included *Nervilia* (one species) and two species of *Epipogium*, finding strong support for a sister relationship between these two genera, which were strongly supported as sister to *Gastrodia* (represented by two species).

Ours is the first study to include representatives from all three genera of Nervilieae, with strong support for relationships among them (Fig. 3c and d), and specifically for the placement of the yet-unstudied *Stereosandra* as sister to (*Epipogium*, *Nervilia*). Chase et al. (2015), in the most recent orchid classification system, recognized two subtribes within Nervilieae: Nerviliinae, composed solely of *Nervilia*, and Epipogiinae Schltr., composed of the fully mycoheterotrophic *Epipogium* and *Stereosandra*. Our findings are at odds with this circumscription at the subtribal level, suggesting that Epipogiinae may be paraphyletic if *Stereosandra* is included within it. However, in our view it seems unnecessary to designate subtribes for a tribe with three genera. The fully mycoheterotrophic genus *Silvorchis* J.J. Sm., represented by a single, presumably extinct species collected once in Java (Smith 1907) was placed within Nervilieae in an earlier treatment (Pridgeon et al. 2009). A second species, *Silvorchis vietnamica* Aver., Dinh & K.S. Nguyen, was described from Vietnam in 2018, as a single-site endemic (Averyanov et al. 2018, Samigullin et al. 2024). The new species closely resembles *Vietorchis*, another fully mycoheterotrophic genus recently demonstrated based on plastome data to belong to subfamily Orchidoideae, though the authors make distinctions between the two genera (Samigullin et al. 2024). *Silvorchis* shares sectile pollinia (divided into subunits larger than tetrads) with *Epipogium*, *Nervilia*, and *Stereosandra*, but this character state is also observed among the Orchidoideae (Freudenstein and Rasmussen 1997, Olędrzyńska et al. 2016). Chase et al. (2015) did not recognize *Silvorchis* (or *Vietorchis*) as members of Nervilieae, nor Epidendroideae for that matter. The phylogenetic affinities of *Silvorchis* thus remain to be addressed, specifically evidence of membership in Nervilieae and how this would affect interpretation of the history of mycoheterotrophic evolution in the tribe.

### Analysis of selection

The finding of significantly relaxed selection for five housekeeping genes is unsurprising, given that several genes within this category have been deleted or are pseudogenes in *Stereosandra* (Figs. 2, 3, 5). Four of these are involved in translation. However, no other plastid genes encoding ribosomal proteins have been lost or pseudogenized in *Stereosandra*, suggesting they may produce functional products, and that translation may be under some level of functional constraint. By contrast, *Epipogium* has lost six ribosomal protein genes and all but five (*E. aphyllum*) and seven (*E. roseum*) tRNA genes (Fig. 3). *Stereosandra* has lost or bears pseudogenes for seven tRNA genes, and these same genes are missing in *Epipogium*.

The *matK* gene is of particular interest due to hypothesized interaction with plastid genes containing Group IIA introns (Zoschke et al. 2010). *matK* displays the strongest signal of relaxed selection among those for which relaxation was detected in *Stereosandra* (Fig. 5), and it is intuitive that four of the seven genes with which *matK* interacts have been lost or pseudogenized. The 5′ exon of *trnK-UUU* is missing in *Stereosandra* (Fig. 1). The *matK* gene resides within the *trnK-UUU* (Group IIA) intron, and so the MatK protein is responsible for removal of both the *trnK-UUU* intron and its own reading frame. *matK* is completely missing in *Epipogium* (Fig. 3; Supplementary Figure S1; (Schelkunov et al. 2015)), but both species of *Epipogium* as well as *Stereosandra* contain putatively functional copies of *clpP* and *rpl2*, both of which contain Group IIA introns. As far as is known, there are no examples among mycoheterotrophic orchids in which *matK* is lost but all Group IIA introns, and the genes that contain them, are putatively functional (Supplementary Figure S2; Graham et al. 2017).

### Ancestral state reconstruction

Stochastic character mapping of trophic mode (leafy, autotrophic vs. leafless, fully mycoheterotrophic) shows evidence with high probability that *Epipogium* and *Stereosandra* evolved independently from leafy hypothetical ancestors (Fig. 6). This adds a previously unknown instance of the transition from autotrophy to heterotrophy in plants (Barrett et al. 2014, 2019, 2025, Feng et al. 2016, Banerjee and Stefanović 2020). Of course, the caveats to interpretation of character state evolution here are topological uncertainty the assumption of irreversible character state changes (i.e. leafless to leafy). Without a strongly supported hypothesis of relationships among the three genera of Nervilieae, it would be easy to assume that *Epipogium* and *Stereosandra* share a sister relationship, and that mycoheterotrophy evolved once in their common ancestor, which is likely the reason both genera are treated in the subtribe Epipogiinae (Chase et al. 2015). Reliance upon plastid genomes alone does not provide support for our conclusions, and our study provides an important example of the benefits of integrating knowledge across genomes.

## Conclusions

By providing phylogenomic resolution within the subtribe Nervilieae, coupled with plastid genome analysis, we demonstrated two independent transitions to heterotrophy from leafy ancestors in the orchids. We provide the first genetic/genomic data for the poorly understood *Stereosandra javanica*, which we consider a ‘transitional’ mycoheterotroph compared to the related *Epipogium*. We emphasize the importance of taxon sampling and the inclusion of data from multiple genomes in analysing the transition to heterotrophy in orchids and other clades that contain parasites. While issues of missing data and long branch attraction using plastid data in clades containing heterotrophs are well documented, we encourage the continued sequencing and analysis of these genomes. They are highly informative and useful ‘markers’ of ecological, genomic, and physiological importance, especially when used in combination with approaches such as stable isotopic profiling. Our findings add to a growing body of data on the evolution of mycoheterotrophy in orchids (and parasitism in plants broadly), which improves our understanding of the evolutionary history of these intriguing plants.

## Supplementary Material

plag002_Supplementary_Data

## Data Availability

Plastid genome annotations can be found at the US National Center for Biotechnology Information (NCBI) under GenBank accession numbers PV739432, PV763472, and PV763473. Raw data can be found at the NCBI Sequence Read Archive under BioProject PRJNA1262965. Code used for data analyses can be found at GitHub: https://github.com/barrettlab/2025_Nervilieae. All datasets can be found at Zenodo: https://doi.org/10.5281/zenodo.15866099
