# Supplementary material for: Mycoheterotrophy and plastid genome evolution in the early-diverging epidendroid orchid tribe Nervilieae: independent transitions in Epipogium and Stereosandra
Source: AoB Plants. 2026 Jan 16;18(1):plag002. doi: 10.1093/aobpla/plag002 (PMC12888386; doi:10.1093/aobpla/plag002)
Supplement: plag002_Supplementary_Data [file plag002_supplementary_data.pdf]

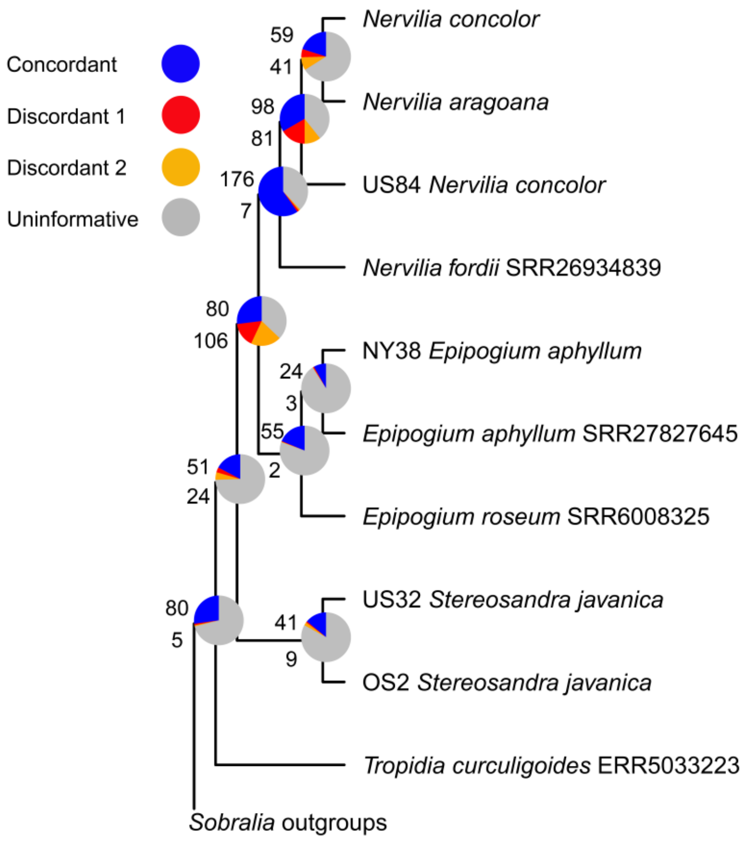

Fig. S1. Gene tree concordance and conflict among topologies for the WASTRAL species tree analysis (corresponding to Fig. 4F). Pie charts indicate the proportion of gene trees concordant (clue), discordant (red, orange for the two discordant quartets), or uninformative (gray) for each node. Numbers on branches are the total number of concordant (above) and discordant resolutions (below) for each node.

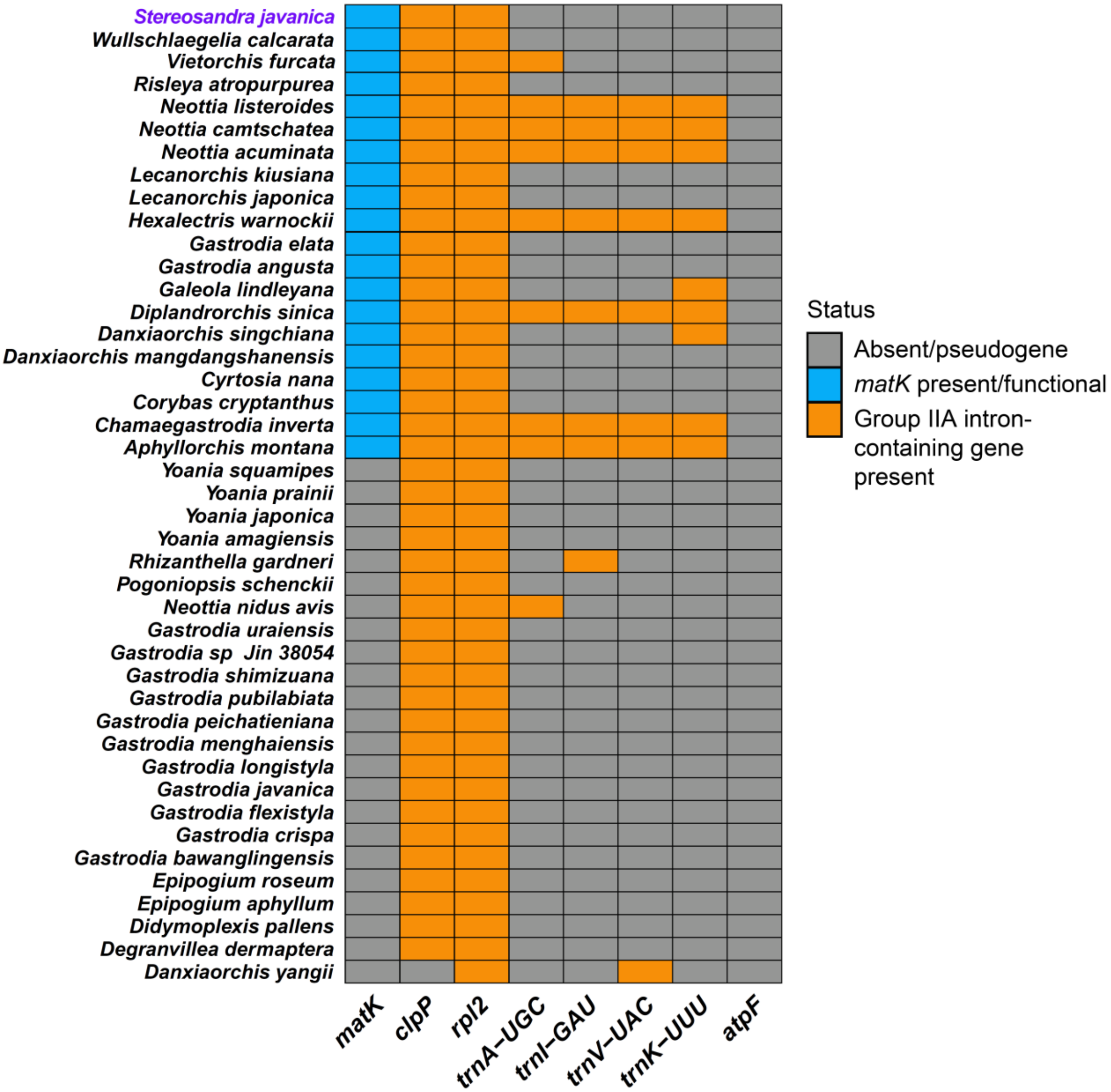

Fig. S2. Presence and absence of *matK* and Group IIA introns in mycoheterotrophic orchids.

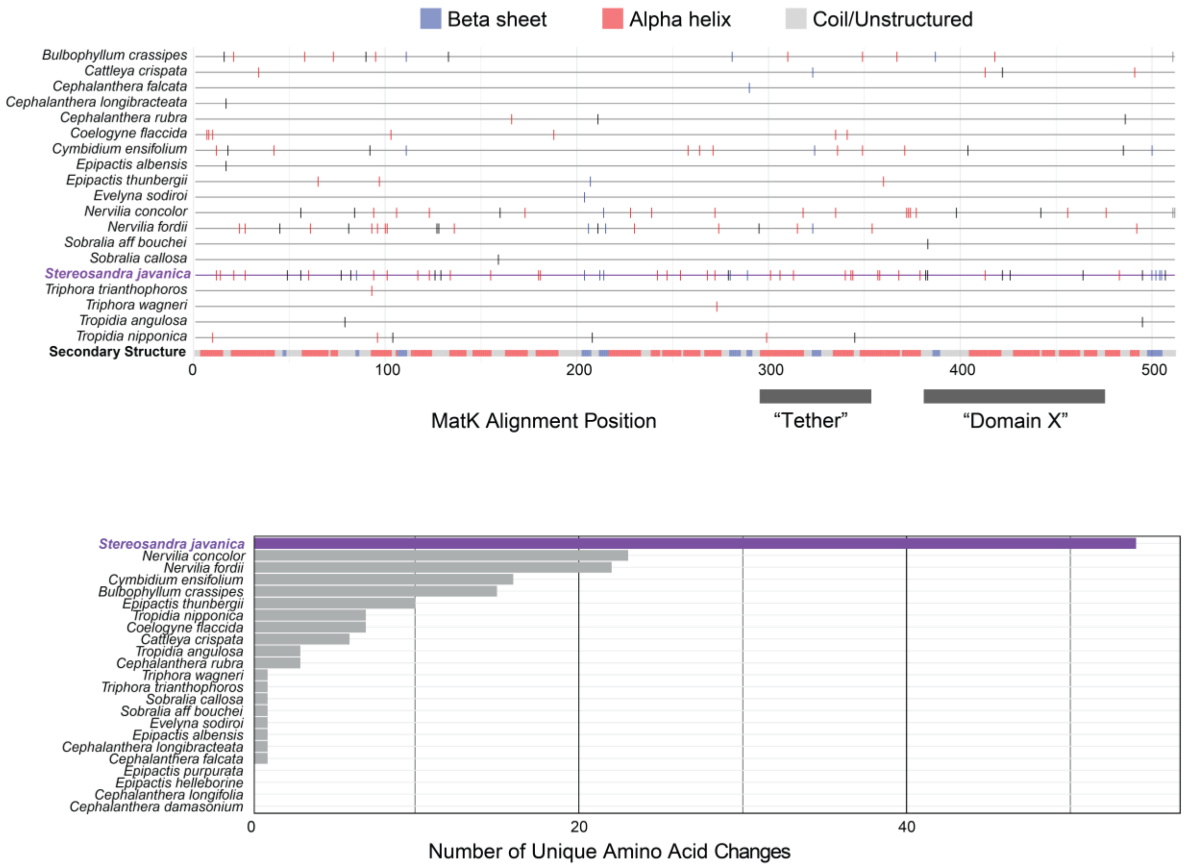

Fig. S3. Above: Unique amino acid changes in the predicted MatK protein for members of orchid tribe Nervilieae and other representative members of the subfamily Epidendroideae. Below: Total numbers of unique MatK amino acid changes.

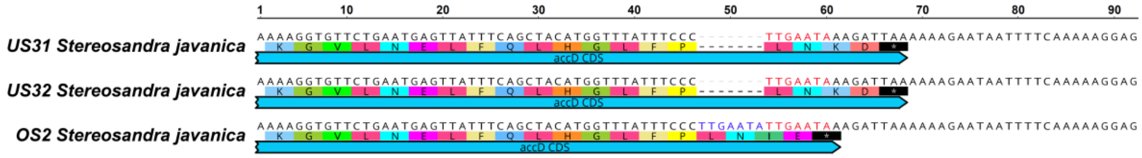

Fig. S4. The 3' region of the *accD* gene among three sequenced accessions of *Stereosandra javanica*.
